# Ecosystem responses to warming and watering in typical and desert steppes

**DOI:** 10.1038/srep34801

**Published:** 2016-10-10

**Authors:** Zhenzhu Xu, Yanhui Hou, Lihua Zhang, Tao Liu, Guangsheng Zhou

**Affiliations:** 1 State Key Laboratory of Vegetation and Environmental Change, Institute of Botany, Chinese Academy of Sciences, Beijing 100093, China; 2 State Key Laboratory of Severe Weather, Chinese Academy of Meteorological Sciences, Beijing 100081, China

## Abstract

Global warming is projected to continue, leading to intense fluctuations in precipitation and heat waves and thereby affecting the productivity and the relevant biological processes of grassland ecosystems. Here, we determined the functional responses to warming and altered precipitation in both typical and desert steppes. The results showed that watering markedly increased the aboveground net primary productivity (ANPP) in a typical steppe during a drier year and in a desert steppe over two years, whereas warming manipulation had no significant effect. The soil microbial biomass carbon (MBC) and the soil respiration (SR) were increased by watering in both steppes, but the SR was significantly decreased by warming in the desert steppe only. The inorganic nitrogen components varied irregularly, with generally lower levels in the desert steppe. The belowground traits of soil total organic carbon (TOC) and the MBC were more closely associated with the ANPP in the desert than in the typical steppes. The results showed that the desert steppe with lower productivity may respond strongly to precipitation changes, particularly with warming, highlighting the positive effect of adding water with warming. Our study implies that the habitat- and year-specific responses to warming and watering should be considered when predicting an ecosystem’s functional responses under climate change scenarios.

Global air temperatures are expected to show continuous increases through the end of this century, mainly due to the ongoing elevation of greenhouse gases, such as CO_2_, and land use changes, such as deforestation^1^,^2^. The frequency and severity of extreme climatic events will be increased, further threatening terrestrial ecosystem stability^3^,^4^. The combined impacts of climate change factors, including intensified heat waves and abnormal precipitation, have already led to the functional and structural degradation of particularly vulnerable terrestrial ecosystems. The damaged areas include the grassland and desert ecosystems of semiarid and arid areas^5^,^6^,^7^,^8^, where the results appear as accelerated desertification^9^,^10^, biodiversity loss^5^,^6^, and altered carbon balance^11^,^12^. Elevated temperature may dry the soil^9^,^13^,^14^, leading to water stress, further aggravating ecological vulnerability and sensitivity and diminishing resilience to rapid degradative changes in these regions^4^,^15^,^16^. As a result, the addition of water can enhance ecosystem function by increasing productivity and photosynthetic capacity^17^,^18^,^19^,^20^,^21^ and improving the carbon balance^22^, thereby alleviating the adverse effects of climate warming^7^,^16^,^22^ and even enhancing the positive effects of warming^23^. Moreover, altered precipitation patterns in terms of frequency, intensity, legacy, and pulse size, as well as seasonal changes in precipitation, can also markedly affect these arid ecosystems’ functions, such as carbon flux^24^,^25^,^26^,^27^, water exchange^25^, and plant physiological status^24^,^28^. Nevertheless, the expected intensification and increasing frequency of extreme climate change events, encompassing high summer temperatures and increased variability in precipitation, threaten sustainable development via both biophysical and socioeconomic factors in semiarid and arid regions^2^,^20^,^29^.

Aboveground plant primary production and microbial activities underground are two critical proxies for ecosystem function^1^,^30^. Increased precipitation may affect the soil microbial status, generally by increasing the microbial biomass and activity and altering the microbial community composition^31^,^32^,^33^. Microbes in terrestrial ecosystems also respond strongly to climate change factors such as water status and temperature^30^,^34^,^35^,^36^,^37^, indicating that the integration of microbial activities into ecosystem processes might be required for an effective and appropriate assessment of the terrestrial ecosystem’s carbon, water, and energy balances under climate change^30^,^32^,^38^. Climate warming and increased variability in precipitation may together regulate the function and structure of ecosystems in temperate savannas, possibly causing a shift from grass-dominated to woody-dominated vegetation^6^. Many studies have reported the effects of heat waves^39^,^40^,^41^, altered precipitation^22^,^42^,^43^,^44^, and their combination^40^,^45^ on plant growth, carbon exchange, and productivity. In contrast, only a relatively small number of investigations have examined the response of microbial activity to climate change. These studies show that the microbial biomass and activity decrease with substantial warming^31^,^46^,^47^, whereas they increase with watering^31^,^36^,^48^. Extensive studies on the relationship between aboveground and belowground functional processes in these major terrestrial ecosystems are largely lacking, and such studies are urgently needed for the assessment of ecosystem responses to climate change and their feedback^20^,^49^,^50^.

Grasslands provide many essential benefits for humans, including forage for livestock, food, biodiversity, carbon storage, and recreation. Grasslands currently cover more than 40% of the Earth’s land surface^51^,^52^. By contrast, drylands, which include arid, semiarid, and dry subhumid areas with scant precipitation (aridity index < 0.65), cover approximately 41%^9^,^14^,^50^. In China, grasslands account for over 40% of the total land area, making China’s the third largest grassland ecosystem in the world^52^,^53^. The largest grassland area in China lies in semiarid and arid regions, mainly in Inner Mongolia, and is a representative part of the central Eurasian steppe region that stretches 8000 km from the northeastern part of Northern China and Mongolia to its western end in Hungary. This region is the largest contiguous grassland area in the world^53^. China’s grassland region, however, is facing severe degradation; those parts that show degradation that is greater than moderately severe now accounts for more than half of the total area, with degradation mainly due to improper land use (e.g., overgrazing) and adverse climatic changes (e.g., altered precipitation and heat)^7^,^22^. A few studies have indicated that the effects of altered precipitation and warming are comparable, particularly as combined factors in most vulnerable arid areas^6^,^54^. Furthermore, studies have reported on the relationships between aboveground and belowground processes^51^ in different grassland ecosystem types^14^,^20^,^53^. Overall, the effects of climatic change may depend on habitats^14^,^20^,^53^. In the present study, we focus on the effects of altered precipitation and warming in a typical and a desert steppe; the former is located further to the east and is characterized by greater precipitation, productivity, and diversity than the latter^37^,^41^,^53^,^54^. To our knowledge, this is the first study that compares the effects of climate change on ecosystem functions, including aboveground and belowground processes, between these two steppe types by using a field-warming facility with three watering levels in both drier and wetter years.

Understanding the adaptive capacity of ecosystems to buffer the negative effects of climate change *in situ* is critical for more precise predictions about and better management of vulnerable ecosystems^29^,^55^,^56^. Our aim is to quantify the singular and combined effects of warming and watering on both aboveground and belowground ecosystem processes (plant primary production, photosynthetic potential, soil respiration (SR), and microbial activity). We will explore these processes in two contrasting vegetation types. Two hypotheses were tested: (1) an interaction exists between warming and precipitation, with precipitation changes being more important than temperature; (2) the warming effect is greater in the desert than in the typical steppes and greater in a drier year than in a wetter year, showing that the effects of climatic change strongly depend on ecosystem type and differences in weather occurring between years.

## Results

### Effectiveness of warming manipulation

A dramatic temperature rise of *ca*. 4.1 °C (day/night temperature rise of 4.10/4.19 °C) was found at the soil surface layer (0–5 cm soil depth) without plant cover, indicating that it warmed more during the night; a marked soil moisture reduction of 18.7% at 0–20 cm depth was observed, indicating that the warming led to a soil water deficit in the arid ecosysems^57^.

### Effects of watering and warming on community productivity

**Figure 1:**
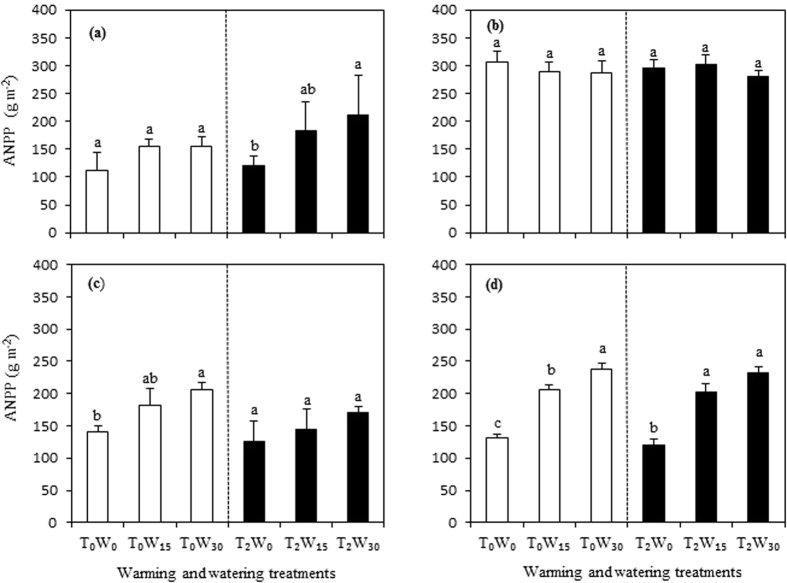
Effects of warming and watering on the annual aboveground net primary productivity (ANPP) in typical (**a,b**) and desert steppes (**c,d**) in 2011 (**a,c**) and 2012 (**b,d**). The dark and light bars represent warming and no warming treatments, respectively. Based on the one-way ANOVA, different lower case letters indicate differences between water treatments at the same temperature with an LSD multiple comparison test, whereas * indicates differences between warming and no warming within a watering treatment at *P* < 0.05. A three-way ANOVA between temperature, precipitation, and ecosystem type is shown in Tables S1 and S8. T_0_W_0_, T_0_W_15_, and T_0_W_30_ denote ambient temperature (T_0_) with normal precipitation (W_0_), plus 15% precipitation relative to average annual precipitation over the past 30 years (1978–2007, W_15_), and plus 30% precipitation (W_30_), respectively, whereas T_2_W_0_, T_2_W_15_, and T_2_W_30_ denote warming (T_2_) with normal precipitation (W_0_), plus 15% precipitation (W_15_), and plus 30% precipitation (W_30_), respectively. Vertical bars represent the SE of the mean (n = 3–4).

In the typical steppe ecosystem in a given drier year (i.e., 2011), the annual aboveground net primary productivity (ANPP) significantly increased by 76.6% with the addition of 30% more water under warmer temperatures (*P* < 0.05), with no effect at ambient temperature (Fig. 1a). No detectable effects of watering were found in a given wetter year (i.e., 2012, Fig. 1b). An addition of 15% more water did not affect the ANPP in the ecosystem in either year. The ANPP increased with temperature in the drier year but not in the wetter year, indicating a lower precipitation in the given drier year had a greater positive warming effect, particular under additional water treatments (Fig. 1a,b). The results showed that the plus 30% watering treatment at warmer temperature had a significant increase in ANPP in the drier year, but not in the wetter year, indicated that the lower precipitation in the drier year (i.e., the given year, 2011) enhanced, while greater precipitation in the wetter year weakened the additional water effects. It is demonstrated the dramatically distinct effects of climatic factor treatments between the two years due to the great variations in the precipitation level of a given year.

In the desert steppe, the ANPP significantly increased by 45.3% with the addition of 30% more water at ambient temperature (*P* < 0.05), with no effect at a warmer temperature or with the addition of 15% more water in the given drier year (Fig. 1c). In the wetter year, the ANPP significantly increased by 79.4% and 94.0% with the addition of 30% more water at ambient and warming temperatures, respectively (*P* < 0.05). These results above showed, in the desert steppe ecosystem, plus water treatment increased more ANPP in the wetter year than in the drier year. There also were significant increases with the addition of 15% more water at both temperatures in the wetter year but not in the drier year (Fig. 1c,d). General decreases in the ANPP were found with warming in the desert steppe ecosystem in both years (−15.1% in the drier and −3.5% in the wetter years across all watering treatments; Fig. 1c,d). Overall, a significant difference in the ANPP was found only at ambient temperature in the desert ecosystem in the wetter year between the additions of 15% and 30% more water treatments (Fig. 1d).

**Figure 2:**
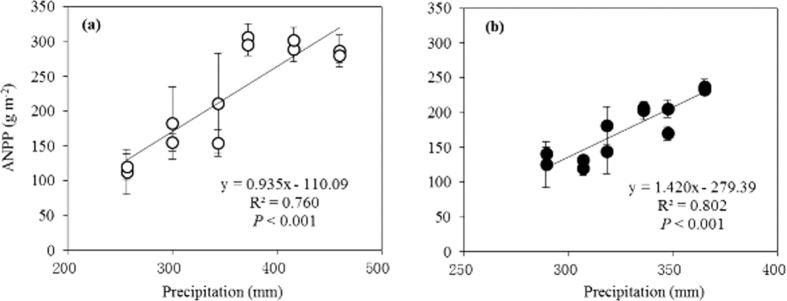
Relationships of the aboveground net primary productivity (ANPP) with annual precipitation in typical (**a**) and desert steppes (**b**).

We found significant linear relationships between the ANPP and precipitation at both experimental sites, with a steeper slope for the desert steppe (*P* < 0.001; Fig. 2). Three-way ANOVAs (Tables S1 and S8) indicated that watering and the interaction of ecosystem type with warming had significant effects on the community productivity (*P* < 0.05) in the given drier year. The ecosystem type, watering, and the interaction of type with watering were significant in the given wetter year. Therefore, climate factor effects were strongly dependent on ecosystem type and yearly variations, and precipitation patterns played a vital role in the productivity response to climate change in semiarid and arid areas during the two years.

### Changes in the photosynthetic activity and soil respiration (SR)

**Figure 3:**
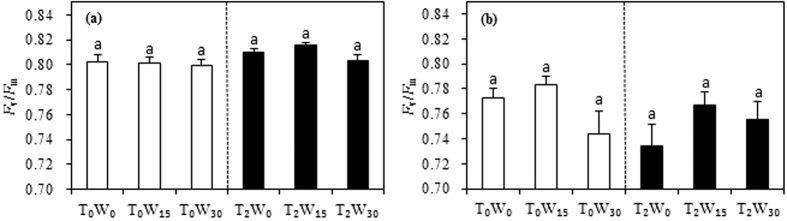
Effects of warming and watering on the maximum photochemical efficiency of photosystem II (*F*_v_/*F*_m_) in typical (**a**) and desert steppes (**b**) at the growth peak during 2011. The dark and light bars represent warming and no warming treatments, respectively. A three-way ANOVA between temperature, precipitation, and ecosystem type is shown in Tables S2 and S8. For abbreviations of the treatments see Fig. 1. Vertical bars represent the SE of the mean (n = 24–56).

As shown in Fig. 3, the maximum photochemical efficiency of photosystem II (*F*_v_/*F*_m_)—a proxy of photosynthetic capacity with non-intrusive measurement—was not significantly affected by watering and warming in either typical or desert steppes. The greatest value was obtained in the typical steppe with the treatment consisting of the addition of 15% water with warming (Fig. 3a), but the greatest value was found in the desert steppe for the addition of 15% water with no warming (Fig. 3b). A slight increase occurred with warming in the typical steppe, whereas a decrease was observed in the desert steppe, indicating opposite effects on the photosynthetic capacity of the dominant species for the two ecosystem types. *F*_v_/*F*_m_ was 6.6% higher in the typical steppe than in the desert steppe (0.81 vs. 0.76, *P* < 0.001), with a minimum under warming with no watering in the desert ecosystem type. A three-way ANOVA indicated significant effects on *F*_v_/*F*_m_ from the ecosystem type or watering as a single factor and an interaction between type and temperature (*P* < 0.05, Tables S2 and S8).

**Figure 4:**
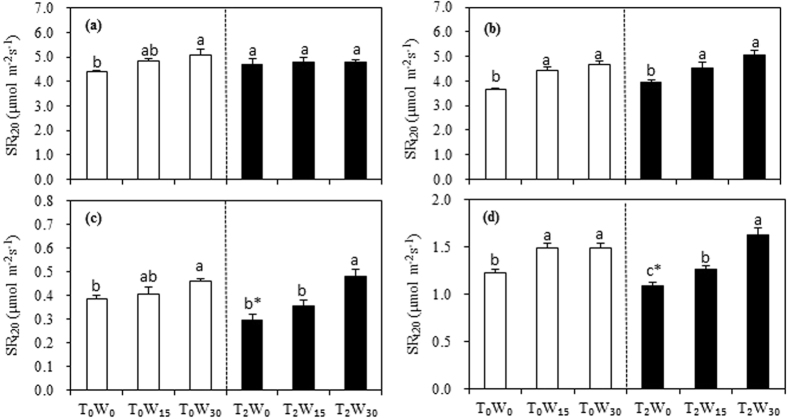
Effects of warming and watering on the soil respiration rate normalized at 20 °C (SR_t20_) in typical (**a,b**) and desert steppes (**c,d**) at the growth peak in 2011 (**a,c**) and 2012 (**b,d**). The dark and light bars represent warming and no warming treatments, respectively. Based on the one-way ANOVA, different lower case letters indicate differences between water treatments at the same temperature with an LSD multiple comparison test, whereas * indicates differences between warming and no warming within a watering treatment at *P* < 0.05. A three-way ANOVA between temperature, precipitation, and ecosystem type is shown in Tables S3 and S8. Vertical bars represent the SE of the mean (n = 15–30). For abbreviations of the treatments see Fig. 1. Note the differences in the y-axis scales.

Soil respiration rate, which is a key parameter of belowground processes, normalized at 20 °C (SR_t20_) during the daytime, increased with watering, and was significantly affected by the addition of 30% water at both temperatures and in both ecosystems (except during 2011 in the typical steppe with warming) (*P* < 0.05; Fig. 4a–d). Warming did not significantly affect SR_t20_ in the typical steppe, but a significant depression occurred when the desert steppe ecosystem was exposed to warming under ambient rainfall conditions (*P* < 0.05, Fig. 4c,d), indicating that warming might exacerbate drought limitations of the SR in more arid areas. Generally, greater watering effects on SR_t20_ were observed in the desert steppe, particularly at the higher temperatures (Fig. 4c,d), regardless of precipitation levels in a given year. SR_t20_ was 7.6 times higher in the typical steppe than in the desert steppe (*P* < 0.001), whereas Q_10_ was 11.0% higher in the desert ecosystem (1.59 vs. 1.43), as determined by the best-fit exponential equations (Fig. S2), again implying a higher vulnerability to climate change in the barren desert steppe. Based on a three-way ANOVA, type and watering as single factors produced significant effects (*P* < 0.05) in the given drier year, and the three factors and their interactions all had significant affects in the given wetter year (*P* < 0.05) (Tables S3 and S8).

### Changes in the carbon and nitrogen components in soil

As shown in Table 1, water application and warming produced significant effects on the soil microbial biomass carbon content (MBC) in the given drier year with a lower precipitation in the typical steppe (*P* < 0.05), with a marked increase resulting from either additional precipitation or rising temperature and a maximum effect demonstrated with the T_2_W_15_ (warming with plus 15% watering) treatment. Watering and warming, however, did not lead to marked changes in the soil total organic carbon (TOC), ammonium-N (NH_4_^+^-N), and nitrate-N (NO_3_^−1^-N) contents. However, no significant changes were found in the given wetter year (Table 1, upper part). In the desert steppe, watering produced significant increases in the TOC and the MBC in either given drier or wetter years, but warming had no significant effect (Table 1, lower part). Increases in the NH_4_^+^-N and NO_3_^−1^-N appeared with increasing precipitation. A marked decrease in the NO_3_^−1^-N occurred with warming under a lower precipitation level during the drier year, but no significant changes were detected for the inorganic nitrogen components in the wetter year in the desert ecosystem, which had generally lower levels. Three-way ANOVAs revealed significant effects from the ecosystem type for the TOC in both years; watering, and type × temperature for the MBC in the given drier year; the type for the NH_4_^+^-N in the given wetter year; and the type for the NO_3_^−^-N in both years (Tables S4–8).

### Relationships among biological processes

**Figure 5:**
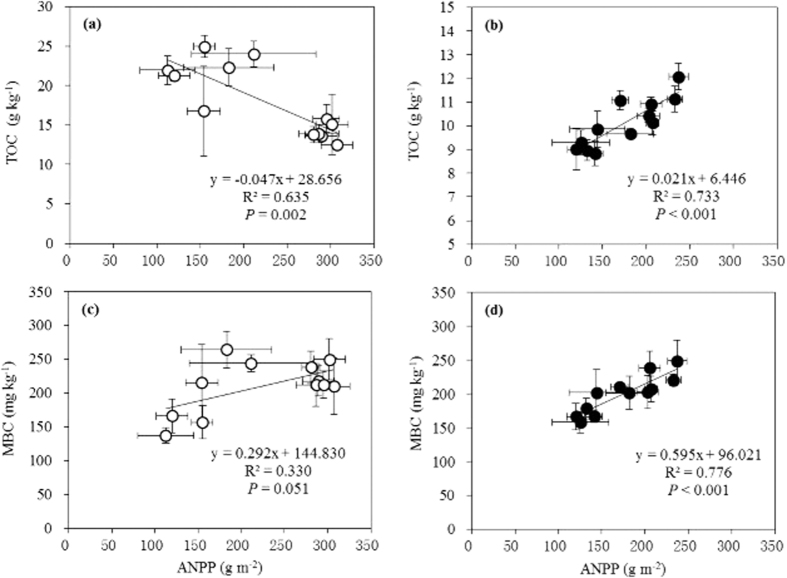
Relationships of ANPP with soil TOC and MBC in the typical (**a,c**) and desert (**b,d**) steppes. For abbreviated details, see Table 1. Note the differences in the y-axis scales of the upper panels.

**Figure 6:**
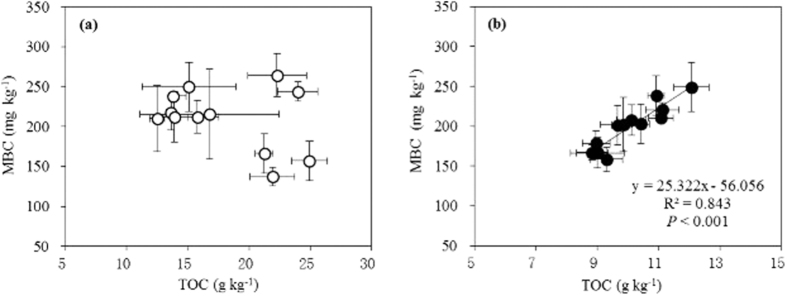
Relationships of soil TOC and MBC in typical (**a**) and desert (**b**) steppes. For abbreviated details, see Table 1. Note the differences in the x-axis scales.

The relationships of the soil nutrient traits and microbial activities with precipitation changes were tested in the typical steppe, and showed positive effects for the MBC (*P* = 0.058) and the NO_3_^−1^-N concentrations (*P* = 0.001) but negative effects for the TOC (*P* = 0.002) and the NH_4_^+^-N (*P* = 0.031, Fig. S3). The desert ecosystem showed positive and significant relationships for precipitation with both the TOC and the MBC (*P* < 0.001), but no significant relationships between inorganic nitrogen components and precipitation (Fig. S3). The ANPP was negatively correlated with the TOC (*P* = 0.002; Fig. 5a) and weakly positively correlated with the MBC in the typical steppe (*P* = 0.051; Fig. 5c), irrespective of the great scattered points. The remarked variations between the two years’ weather patterns can be mainly explained: The ANPP had greater values but with no significant responses to watering treatments in 2012 (a wetter year). However, in this given year, there were lower values in TOC and greater values in MBC, together leading to the more scattered distributions (Fig. 5a,c). However, the ANPP was strongly positively correlated with both the TOC and the MBC in the desert steppe (R^2^ > 0.70, *P* < 0.001; Fig. 5b,d). A significant relationship between the TOC and the MBC was found in the desert steppe only (*P* < 0.001; Fig. 6).

**Figure 7:**
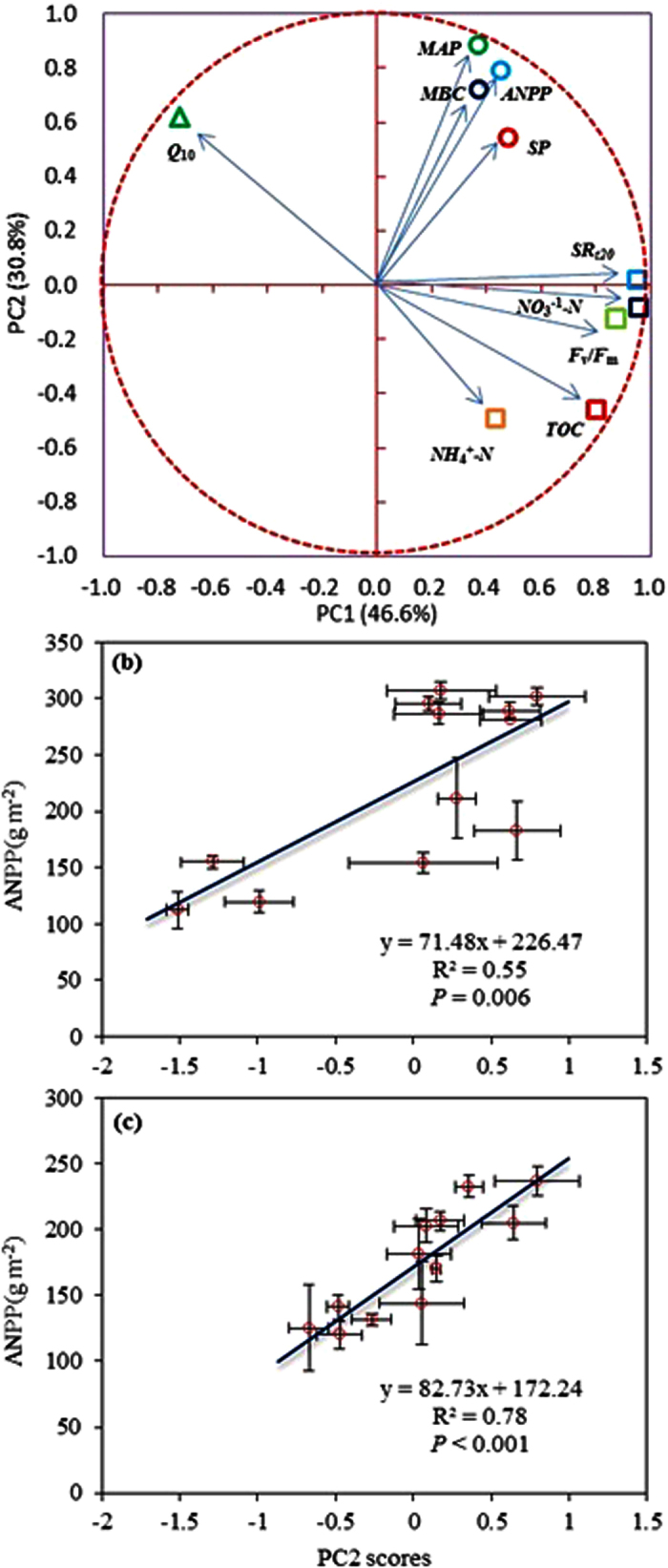
Loadings from the first two principal components (PCs) derived from principle component analysis (PCA) for all parameters (**a**); the relationships of annual aboveground net primary productivity (ANPP) with secondary principal component scores (PC2)—only summarizing several belowground process features—in typical (**b**) and desert steppes (**c**). *F*_v_/*F*_m_, maximum photochemical efficiency of photosystem II; MAP, mean annual precipitation; MBC, microbial biomass carbon; *Q*_10_, soil respiration rate (SR) sensitivity to temperature; SP, growth seasonal precipitation; SR_T20_, SR at 20 °C; TOC, soil total organic carbon.

A principal component analysis (PCA) was conducted to determine the multivariate pattern of the treatments factors’ effects (Fig. 7a). The first two principal components (PCs) accounted for 77.4% of the total variables. The loadings of community production, MBC, and precipitation were distributed in quadrant I, with *Q*_10_ in quadrant II, while the proxies representing belowground process traits were gathered in quadrant IV (Fig. 7a). Furthermore, the primary and secondary ordination axes (PC1 and PC2) were extracted by another PCA that only included the belowground process properties; these axes explained 75.5% of the total variations belowground. Interestingly, the PC2 was significantly associated with the ANPP, which was stronger in the desert steppe (R^2^ = 0.78, *P* < 0.001) than in the typical steppe (Fig. 7b,c; R^2^ = 0.55, *P* = 0.006), again highlighting the tighter linkage between above- and belowground processes in the desert ecosystem.

## Discussion

Continuing climate warming may constrain the ecosystem functions of drylands, whereas increased precipitation might alleviate the negative effect of warming^14^,^31^. Although our understanding of the responses to climate change in arid ecosystems is improving, *in situ* manipulations of warming with precipitation alteration are still scant^14^,^32^. The present field experiments investigated ecosystem functional responses to warming and increased precipitation in both typical and desert steppes over two consecutive growing seasons. A dramatic temperature rise was observed with the field-warming facility with a greater warming occurring during the night. This warming process was consistent with the climate change prediction that global surface temperature is expected to elevate by 0.3–4.8 °C by the end of 2100^2^, with greater nocturnal warming in terrestrial environments^58^. This asymmetric warming produces different effects on terrestrial ecosystem functions^59^, such as limited plant growth of a steppe grass^60^, decreased grassland ANPP in North China^61^, less response in terms of phenological bud break of *Picea mariana* seedlings^62^, and no significant effect on wheat growth and yield in North China^63^. Our warming manipulation in a natural field can also mimic the effects of the predicted climate change scenario.

Our current results showed that the effect of warming on the ANPP increased with additional precipitation in the desert ecosystem in both the wetter and drier years. However, in the typical steppe ecosystem, productivity responded to warming and additional precipitation only in the given drier year, indicating that the interactive effects of warming and precipitation may depend on the ecosystem type and the annual precipitation level of a given year. Precipitation rather than temperature was the primary driver of the ANPP and belowground processes in these systems. Our results additionally showed that the belowground processes were associated with the ANPP more strongly in the desert steppe than in the typical steppe. Thus, the desert steppe may have a more sensitive response to precipitation change with warming, particularly in a given drier year; this indicates that the responses depend on ecosystem type and precipitation pattern of a given year, which must be considered when forecasting an ecosystem’s functional responses to future climatic change.

### Effects of watering and warming on productivity

Water status plays an important role in the functional response to climate change in grassland and desert ecosystems in semiarid and arid regions^21^,^22^,^64^. For example, additional precipitation can increase the ANPP of grassland and desert ecosystems, particularly in arid areas^17^,^22^,^44^. Water application may exaggerate the positive effects of warming on plant growth^7^,^65^,^66^. A significant positive effect from increased precipitation was observed on the ANPP under warmed conditions, but only in drier years^67^ or following a four-year long-term warming^65^. In the current experiment, we also found a significant effect of adding water in the drier year with lower precipitation relative to the wetter year (Fig. 1). This can be explained by that a great stimulation occurs due to water addition often under a water deficit environment compared to ample water status^19^,^24^,^67^. As suggested by many investigators, a higher sensitivity of terrestrial ecosystems to environmental changes such as drought and warming is found in these unproductive regions^14^,^29^,^55^,^68^. Similarly, in our experiment, the desert steppe with lower productivity (ANPP) and photosynthetically physiological activity (*F*_v_/*F*_m_) showed a higher response to precipitation changes, particularly with warming (Fig. 2), highlighting the positive interactive effect of adding water with higher temperatures. Moreover, a report on pasture grassland in central Texas, USA, indicated that a 128 mm increase in precipitation during one summer month could increase the ANPP by 10% (from 333 to 365 g m^−2^) and 1% (from 394 to 398 g m^−2^) in native and exotic communities, respectively^56^, indicating that grassland characteristics, such as community productivity, species composition, and soil type, determine the responses to precipitation. Generally, these grassland ecosystems with barren soil, lower species richness, and lower productivity, may be highly sensitive to precipitation changes^42^,^53^,^54^,^56^, which can be also explained by the current experimental results in the two given years (Figs 1 and 2). Thus, precipitation effects may be predominant in the productivity response to climatic change in arid and semiarid areas, and this response strongly depends on ecosystem type and precipitation of a given year^16^,^56^,^67^,^69^,^70^, as highlighted by the current results.

No statistically significant warming effects were observed on the ANPP in either of the steppe ecosystems, although a decreasing trend appeared in the desert steppe ecosystem (Fig. 1). In the typical steppe, plant biomass and the net ecosystem carbon exchange were not significantly affected by a 1.8 °C warming^71^. However, many observations of *in situ* grasslands have indicated decreases in the ANPP in response to climate warming, particularly in hotter environments^14^,^72^,^65^ and during the summer season^46^,^73^,^74^. For example, a 52% productivity loss occurred in *Lolium perenne* plants exposed to 2 °C warming during summer in Zürich, Switzerland^75^. In contrast, an early meta-analysis including various biomes and several warming strategies^39^ showed that warming significantly increased the ANPP by an average of 19% across 24 sites. General increases in plant biomass were also achieved by warming in a tallgrass prairie in the US Great Plains^76^ and in an upland grassland located in the French Massif Central area^67^. It has been reported that aboveground productivity can be stimulated dramatically by consecutive three-year warming in the tundra^69^ and in an alpine meadow on the Tibetan plateau in China^77^. As reported by Henry *et al*.^78^, a seven-year warming led to a significant increase in the total aboveground biomass only in a year when spring snowmelt was promoted in a grass-dominated temperate old field in Ontario, Canada. Thus, warming often has a negative effect for hotter and drier sites and/or years, and its effect strongly depends on the ecosystem type, the location, and the weather condition of the given year. It is again indicated that the marked dependence on ecosystems and precipitation patterns between the given years must be considered when predicting an ecosystem’s functional responses to climatic warming (Fig. 1)^39^,^77^,^78^.

### Effects of watering and warming on belowground biological processes

The SR increased in response to precipitation but not to warming in the typical steppe ecosystem. This indicates that precipitation is a greater constraint on belowground processes when compared to warming in this system, in agreement with a previous study in the same ecosystem^31^. Moreover, the SR also strongly responds to episodic rainfall^64^, precipitation pulses^12^, water addition gradient^79^, and drying-wetting events^80^. These results again highlight the dominant role of precipitation. In the desert steppe, although the SR also increased with increasing precipitation, it significantly decreased with warming during normal water status—warming may lead to water stress, indicating that an interaction can exist, depending on the ecosystem type^60^,^64^. Thus, for the SR changes, the results concerning the interaction and the major role of watering confirm our first hypothesis (Fig. 4). Similarly, an alpine meadow on the Tibetan plateau responded to field warming with a significantly increased seasonal average SR, which sometimes decreased when water deficits stress occurred^77^. Therefore, the response of soil carbon emissions to climate change may depend on ecosystem type, precipitation patterns, and the interactions between climatic factors^77^,^81^,^82^.

The present study showed that the TOC and the MBC increased with watering in the desert steppe in the both given years in a manner similar to increases in the SR (Table 1). A tight association of the TOC with the MBC occurred in the desert steppe (Fig. 6), indicating that additional water can increase soil and microbial carbon levels and microbial activity in drier environments. This result is consistent with other reports that increased precipitation and thereby an improved soil water status can increase the SR by enhancing root growth and increasing soil microbial activity and organic carbon decomposition, consequently promoting both autotrophic and microbial heterotrophic respirations^12^,^26^,^31^,^32^,^80^,^83^,^84^. Additionally, precipitation patterns such as drying-rewetting cycles can result in significant changes in soil microbial carbon and nitrogen dynamics, ultimately lessening the SR^80^. Moreover, an increase in soil moisture led to a higher soil carbon release but no change in the soil MBC in a Chihuahuan desert grassland^85^.

Most ecosystem models postulate that the microbial decomposition of soil carbon can be stimulated by warming, leading to MBC reduction and TOC depletion^35^,^81^. However, decreases in both microbial enzyme activity and soil microbial biomass have been shown in responses to a 5 °C warming over the long term^38^. An increase in microbial population size occurred in a US tall grass prairie with moderate warming under normal precipitation, whereas a decline was found following a two-year long-term warming treatment with drought^36^, suggesting that warming-induced drought may substantially reduce soil microbial activity^31^,^48^. The temperature sensitivity of microbial decomposition is closely associated with the soil organic carbon quality^86^. No significant effect of warming and its interaction with watering on the TOC and the MBC were observed in our current experiment (Table 1, Tables S4–5). Further research is needed to identify the responses to warming strength or duration and the interaction with precipitation in different ecosystems and the given years.

No systematic effects were found in inorganic nitrogen components, although a generally lower level was found in the desert steppe (Table 1). Generally, nitrate is more stable in soil and is more available to plants, but it is also more sensitive to temperature changes^71^,^87^, which is supported by the marked decrease in the NO_3_^−^−N concentration due to warming in the desert steppe during the given drier year (Table 1)^85^. However, a 1.8 °C warming did not affect the NH_4_^+^ and NO_3_^−^ concentrations^71^. Increases in NH_4_^+^-N and NO_3_^−1^-N appeared with increasing precipitation at the desert steppe ecosystem in the drier year, consistent with a result by Wang *et al*.^88^ that indicated that long-term water addition significantly increased the total extractable inorganic N in the same typical steppe. Thus, this climatic effect on inorganic N dynamics may depend on the ecosystem type and the precipitation of specific given years.

Gestel *et al*.^85^ reported a twofold increase in the MBC with a greater SR by warming, which is a carbon process response, but 16% and 18% reductions occurred in soil NO_3_^−^–N and NH_4_^+^–N availability, respectively, after a three-year warming—an N process response. The combined effects may ultimately lead to the decoupling of the carbon and N balance in belowground biological processes in responses to climatic change^49^,^88^, which also may depend on the ecosystem type and the precipitation of a given year. Soil carbon and nitrogen metabolism may both be affected by the limitations of microbial activity under the more unfavorable environments in the desert steppe, particularly during a given drier year with a low precipitation level (Table 1)^36^. Nevertheless, further studies with relatively long-term experiments are required to solve the related uncertainties^89^.

### Relationships between above- and belowground biological processes

Current terrestrial ecosystem models need to incorporate temperature and/or water effects in aboveground ecosystem functions, such as the ANPP, and belowground processes, such as the SR^44^,^51^,^89^. Differential responses from above- and belowground biota in terrestrial ecosystems to climate change such as shifts in precipitation patterns may alter normal biogeochemical processes^70^. In our experiment in the typical steppe, the SR was stimulated by watering in a similar manner to the ANPP in a given drier year^17^,^44^, but, unlike the SR, the ANPP showed no significant response to warming. In the more arid desert steppe, however, SR changes occurred in response to both warming and watering. These changes occurred in concert with the ANPP, which indicated that the SR response to temperature may be closely associated with the ANPP^90^, again depending on ecosystem type^90^ (Fig. 5). Additionally, soil moisture may regulate the plants’ response to warming; for example, increased soil water availability can enhance the temperature sensitivity of plant growth and respiration^22^,^31^,^91^. Moreover, maintenance of normal productivity and microbial activity may need to be coupled appropriately to sustain ecosystem function^35^, but the underlying mechanism is still unclear^92^,^93^,^94^. Microbial metabolism, including decomposition processes, operates often at a high rate during the active period of plant growth^95^. High productivity, which is generally associated with high plant residues, may promote microbial activity processes by increasing organic matter decomposition^92^,^96^,^97^. However, our analyses indicated that belowground physical and biological traits such as the TOC and the MBC and their integration are more tightly associated with the ANPP in the desert than in the typical steppes (Figs 5 and 7), once again emphasizing the higher sensitivity of the desert steppe ecosystem. Thus, whether and how a the above- and belowground bioprocesses are tightly coupled may largely depend on the ecosystem type and the *in situ* environmental variables^70^,^94^,^98^, as confirmed by the present experiments.

## Conclusions

Our findings are largely consistent with the hypotheses: there are interactive effects of precipitation and climatic warming, depending on the ecosystem type, the precipitation levels of the given years, and certain functional traits (in which the precipitation pattern plays a major role). Warming affected some biological processes, such as the ANPP and the SR, more in the desert than in the typical steppes and in a given drier year more than in a given wetter year. In many arid regions, an increase in evaporative water loss with warming may result in enhanced drought, subsequently constraining the ecosystem functional processes, including plant growth and microbial activity, whereas increased precipitation may exert the opposite effects. Therefore, the expected temperature-driven increases in the process rates may not be obtained and may even shift the direction of the process due to precipitation changes^5^,^7^,^13^,^22^,^99^. The present results with contrasting steppe ecosystems indicated that the desert steppe may more sensitively respond to precipitation changes with warming relative to the typical steppe, implying that a strong dependence on habitat and annual precipitation pattern in a given year should be considered when predicting the functional responses of vegetation to future climatic change. Additionally, in the current experiment, it is noted that our data were collected only from the two ecosystems in the vast temperate grassland during the two consecutive years, and the treatments also included only the three water levels with the two temperatures. The relatively limited data may also limit to test the further lasting effects of long-term field warming and various altered precipitation patterns. Nevertheless, more long-term field experiments with more extensive warming and watering level treatments in various ecosystems are urgently needed to obtain a sound understanding of the aboveground and belowground responses to climate change 3,32,99,100.

## Methods

### Site description

We conducted field-warming experiments in two types of steppes with contrasting traits related to climate and vegetation—a typical steppe and a desert steppe. Typical steppes are found in semiarid climates in temperate zones with an annual precipitation of approximately 350 mm, whereas desert steppes are mostly arid ecosystems with relatively less annual precipitation (often below 250 mm)^53^. The typical steppe site in the present study is eastern, located in Duolun County (42′02″N, 116′17″E, 1324 m a.s.l.), Inner Mongolia, China. This site belongs to a typical temperate zone described by a semiarid continental monsoon climate, with a mean annual temperature (MAT) of 2.26 °C (±0.13, SE) over the last 60 years (1953–2012), a maximum monthly mean temperature of 19.02 °C (±0.14) in July, and a minimum monthly mean temperature of −17.57 °C (±0.29, SE) in January. The mean annual precipitation (MAP) is approximately 380 (±24) mm, with 80% occurring from June to September. The average daily temperature is 16.5 °C during the growing season (*c.*120 d, June–September, 1978–2007). Climate change at this site was indicated by asymmetrical diel warming (0.28, 0.39, and 0.46 °C increases in the daily maximum, mean, and minimum temperatures per decade, respectively, over the most recent 60 years), with a highly variable MAP (CV of 18.8%) (Fig. S1A,C). In 2011, the annual averages of the daily mean, maximum, and minimum temperatures were 2.38, 9.67, and −4.29 °C, respectively; in 2012, the three values were 1.91, 8.88, and −4.46 °C, respectively. The MAP in 2011 and 2012 was 256.1 and 372.3 mm, respectively; 2011 was the drier year with 116.2 mm less precipitation. The soil type was classified as chestnut soil (Calcis-orthic Aridisol) with a mean soil bulk density of 1.31 g cm^−3^. The area was dominated by perennial species, such as *Stipa krylovii* Roshev., *Artemisia frigida* Willd, *Potentilla acaulis* L. and *Cleistogenes squarrosa* (Trin.) Keng^41^.

The second experimental site is to the west, in a desert steppe (41′39″N, 110′20″E; 1409 m a.s.l.) in Damao County, Inner Mongolia, China. In this area, the MAT was 4.21 °C (±0.13) over the last 58 years (1955–2012), with a maximum monthly mean temperature of 21.19 °C (±0.16) in July, a minimum monthly mean temperature of −15.06 °C (±0.32) in January, and an MAP of approximately 256 mm (with 86% occurring during the growth season). The average daily temperature is 18.5 °C during the growing season. Increases of 0.25, 0.38, and 0.54 °C occurred in the daily maximum, mean, and minimum temperatures per decade, respectively, in this desert steppe (1955–2012). The daily minimum temperature was only observed at night, indicating stronger nocturnal warming. The MAP over the past 60 years also shows high variability, with a high CV of 25.4% and a slightly decreasing trend (*P* > 0.05, Fig. S1B,D). In 2011, the annual averages of the daily mean, maximum, and minimum temperatures were 4.45, 11.83, and −1.85 °C, respectively; in 2012, these values were 3.96, 11.27, and −2.19 °C, respectively. In 2011 and 2012, MAPs of 289.1 and 306.8 mm occurred, respectively. The area has a chestnut soil type and a mean soil bulk density of 1.23 g cm^−3^ and is dominated by both annual and perennial species, including *Stipa klemenzii* Roshev., *Neopallasia pectinata* (Pall.) Poljak, *Cleistogenes squarrosa* (Trin.) Keng, and *Artemisia capillaries* Thunb^54^.

### Experimental design

The details of the present experimental design were published in a previous report^54^. Briefly, a randomized complete block design was performed with three precipitation and two temperature treatments in all possible combinations, with four replications of each of the six combinations at each site. Altered precipitation included three treatments: normal precipitation (W_0_), plus 15% precipitation (W_15_), and plus 30% precipitation (W_30_). The total increased precipitation indicated by W_15_ and W_30_ was 15% and 30%, respectively, of the mean growing season precipitations over the past 30 years (291.6 and 193.9 mm in Duolun and Damao, respectively, from 1978–2007). Water applications were performed weekly during the growing season of each year. The temperature manipulation had two treatments: ambient temperature (no warming, T_0_) and high temperature (an expected 4.0 °C warming, T_2_). Each site contained twenty-four plots of 2 × 2 m in area (4 replicates × 6 treatments = 24, and the treatments were 2 temperatures × 3 watering amounts) with a 1 m buffer space between adjacent plots.

A field infrared radiation warming facility—free air temperature increase (FATI)^75^—was used to simulate climate warming effects in the steppe ecosystems, as previously described by Hou *et al*.^54^. An 800 W infrared radiation heater of 1.0 m length (GHT220-800, Sanyuan Huahui Electric Light Source Co. Ltd., Beijing, China) was suspended 1.3 m over the center of each warming treatment plot and was run continuously during the growing season in 2011–2012. A “dummy” heater was also placed over the unheated plots to account for the effects from shading or other factors related to the heating facilities (Plate S1). Warming treatments were conducted in the growing season (early May–late August). Soil temperature (0–5 cm soil depth) and soil moisture (0–20 cm) were monitored using thermocouples (HOBO S-TMB-M006, Onset Computer Corporation, Bourne, MA, USA) and humidity transducers (HOBO S-SMA-M005), respectively. Data were automatically recorded by a logger (HOBO H21-002) every 30 min during the experiments^57^.

### Aboveground net primary productivity (ANPP) measurements

We measured plant productivity at the peak of plant biomass in mid-August in both years in a permanent 1 m^2^ quadrant at the center of each plot. Aboveground plant biomass was measured after carefully clipping each plant 2–3 cm above the soil surface (to mimic land use for mowing management) and then drying in an oven at 65 °C for at least 72 h to obtain a constant dry weight. The ANPP was expressed as g m^−2^ y^−1^.

### Leaf chlorophyll fluorescence determination

Chlorophyll fluorescence was measured predawn in complete darkness using a leaf fluorometer (LI-6400-40, Li-Cor Inc., Lincoln, NE, USA) with an LI-6400F photosynthesis system (LI-6400, Li-Cor Inc.). The minimal fluorescence yield (*F*_0_) was determined with modulated light at a sufficiently low level of 1.0 μmol m^−2^ s^−1^, and the maximal fluorescence yield (*F*_m_) was obtained by a 0.8 s saturating pulse at 8,000 μmol m^−2^ s^−1^. The maximum photochemical efficiency of photosystem II (*F*_v_/*F*_m_) was expressed as (*F*_m_ − *F*_0_)/*F*_m_^101^. Measurements were performed on at least three of the uppermost, fully expanded leaves of three to five dominant species in each treatment. The maximum photochemical efficiency can be an indicator of the response to environmental changes, including watering and temperature^28^,^66^. The data were then averaged to represent plant photosynthetic activity^7^,^102^.

### Measurements of soil physicochemical traits and microbial activities

Soil samples (10 cm in diameter, 0–10 cm soil layer depth) were retrieved with an auger during peak growing season and were then mixed and sieved through a 2 mm diameter mesh. The samples were immediately placed in Ziploc bags for storage in a 4 °C incubator. Soil organic carbon was extracted from soil samples by suspension in 50 ml of 0.5 M K_2_SO_4_ and agitation in an orbital shaker at 120 rpm for 1 h. The filtrate was analyzed with a TOC analyzer (High TOC, Elementar, Hanau, Germany). NH_4_^+^-N and NO_3_^−^-N were extracted by adding 50 ml of 2 M KCl to homogenize the soil sample, and their contents were determined using a flow injection auto-analyzer (FIAstar 5000, Foss Tecator, Hillerød, Denmark). Soil MBC, representative of key microbial activities, was determined using chloroform fumigation extraction^103^,^104^.

### SR rate measurements

The SRs were measured using a LI-8100 portable soil CO_2_ flux system (LI-8100, Li-Cor Inc.) during the peak growing season. Soil surface disturbances were minimized by mounting the chamber on PVC soil collars that were 5 cm in height and 10 cm in diameter and sharpened at the bottom. The soil collars were inserted approximately 2 cm into the soil, and plants in the soil collars were clipped at the soil surface to minimize disturbance by plant respiration one day before measurements; this time was sufficient to allow the SR to completely recover from aboveground disturbance^31^,^76^,^105^. An exponential function was constructed to determine the SR’s sensitivity to temperature change^90^. We selected the SR data for comparison between treatments from 10:00–16:00 during the peak growth period. The SR at 20 °C was used as a standard value to minimize the temperature effects during measurements:


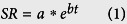



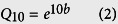



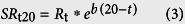


where *a* and *b* are the parameters of the exponential equation, *Q*_10_ represents the SR sensitivity to temperature, and *SR*_t20_ and *R*_t_ are the SR at 20 °C and at the actual temperatures measured, respectively. Parameter *b* was obtained from an exponential equation constructed between the SR and the soil temperature, which was based on data obtained in a 24-hr consecutive measurement so as to create a wide range of temperature changes with a relatively stable level of soil moisture.

### Statistical analyses

The effects of warming, watering, and the interaction of the two for the present experiment were analyzed using SPSS version 20.0 statistical software (SPSS Institute Incorporated, Chicago, IL., USA). For the measured variables, including the ANPP, *F*_v_/*F*_m_, TOC, NH_4_^+^-N, MBC, and SR, we used one-way ANOVAs to test the differences between warming and no warming within a watering treatment and between water treatments at the same temperature with an LSD multiple comparison test. The main effects of temperature, precipitation, ecosystem type, and their interactions were examined using a mixed model of three-way ANOVA. The mean and standard error (±SE) of each treatment are presented in all tables and figures. These relationships of the precipitation changes with ecosystem functional parameters, including the ANPP and the soil nutrition characteristics, were assessed with a linear regression analysis. Finally, the effects on changes in all of the parameters under the combined treatments were examined with a PCA. A second PCA was used to integrate the belowground process traits, including the soil nutrient parameters and the soil microbial activities, which obtained the primary and secondary ordination axes (PC1 and PC2), together representing an integrated belowground process. Thus, the relationships of the principal components with the ANPP can directly assess the association between the belowground and aboveground processes^94^,^106^. Unless otherwise noted, *P* < 0.05 was considered as statistically significant.

## Additional Information

**How to cite this article**: Xu, Z. *et al*. Ecosystem responses to warming and watering in typical and desert steppes. *Sci. Rep.* **6**, 34801; doi: 10.1038/srep34801 (2016).

## Supplementary Material

Supplementary Information

## Figures and Tables

**Table 1:** Effects of warming and watering on the soil total organic carbon (TOC, g kg^−1^), microbial biomass carbon (MBC, mg kg^−1^), NH_4_^+^-N (mg kg^−1^), and NO_3_^−1^-N (mg kg^−1^) in typical (Duolun) and desert (Damao) steppes during 2011 and 2012.

Duolun	2011				2012			
	TOC	MBC	NH_4_^+^-N	NO_3_^−1^-N	TOC	MBC	NH_4_^+^-N	NO_3_^−1^-N
T_0_W_0_	21.93 ± 1.83	137.31 ± 11.30c	9.69 ± 1.92	16.14 ± 2.59	12.48 ± 0.59	210.14 ± 41.86	10.26 ± 1.91	22.02 ± 4.93
T_0_W_15_	24.95 ± 1.44	157.00 ± 24.50a	9.51 ± 1.90	19.75 ± 2.75	13.60 ± 0.41	217.44 ± 22.25	7.75 ± 0.70	22.12 ± 1.63
T_0_W_30_	16.77 ± 5.59	215.58 ± 56.65a	11.36 ± 1.77	20.52 ± 2.41	13.89 ± 1.15	212.14 ± 31.65	9.13 ± 1.53	27.27 ± 5.91
T_2_W_0_	21.25 ± 0.72	166.47 ± 24.71b*	12.12 ± 2.91	19.35 ± 1.49	15.78 ± 1.78	212.30 ± 20.46	8.13 ± 1.53	23.81 ± 1.93
T_2_W_15_	22.30 ± 2.39	264.23 ± 27.02a*	10.38 ± 0.84	22.24 ± 2.67	15.08 ± 3.84	249.84 ± 30.55	7.39 ± 0.50	26.84 ± 4.64
T_2_W_30_	23.99 ± 1.14	244.07 ± 12.38a	7.25 ± 0.25	23.06 ± 2.97	12.48 ± 1.03	238.11 ± 23.95	7.71 ± 0.23	23.32 ± 1.84
**Damao**								
T_0_W_0_	8.84 ± 0.50b	166.90 ± 9.40b	8.10 ± 0.53b	2.28 ± 0.92b	8.97 ± 0.45c	178.68 ± 15.09b	4.58 ± 0.53	0.28 ± 0.10
T_0_W_15_	9.67 ± 0.19ab	201.52 ± 24.72ab	11.54 ± 1.73a	1.74 ± 1.00b	10.12 ± 0.49b	207.30 ± 19.16ab	5.12 ± 0.53	0.95 ± 0.33
T_0_W_30_	10.93 ± 0.27a	238.23 ± 24.84a	9.04 ± 1.14a	3.64 ± 0.88a	12.08 ± 0.56a	248.86 ± 30.58a	4.68 ± 0.53	0.44 ± 0.28
T_2_W_0_	9.31 ± 0.52b	158.34 ± 15.58b	9.85 ± 0.25	1.53 ± 0.68	9.01 ± 0.88b	166.48 ± 19.10b	4.64 ± 0.53	0.73 ± 0.08
T_2_W_15_	9.86 ± 0.75ab	201.88 ± 33.99ab	8.88 ± 0.04	0.94 ± 0.35	10.43 ± 0.28a	202.95 ± 24.36ab	4.97 ± 0.53	0.71 ± 0.17
T_2_W_30_	11.08 ± 0.40a	210.31 ± 3.37a*	8.82 ± 0.48	1.15 ± 0.08	11.13 ± 0.54a	220.70 ± 8.97a	4.88 ± 0.53	0.91 ± 0.31

Means ± SE are presented (n = 3–4).

Different lower case letters indicate differences between water treatments at the same temperature with an LSD multiple comparison test, whereas * indicates differences between warming and no warming within a watering treatment at *P* < 0.05. Three-way ANOVAs on the interactions between temperature, precipitation, and ecosystem type are shown in Tables S4–8. T_0_W_0_, T_0_W_15_, and T_0_W_30_ denote ambient temperature (T_0_) with normal precipitation (W_0_), plus 15% precipitation relative to average annual precipitation over the past 30 years (1978–2007, W_15_), and plus 30% precipitation (W_30_), respectively, whereas T_2_W_0_, T_2_W_15_, and T_2_W_30_ denote warming (T_2_) with normal precipitation (W_0_), plus 15% precipitation (W_15_), and plus 30% precipitation (W_30_), respectively.
