# Supplementary material for: Ecosystem responses to warming and watering in typical and desert steppes
Source: Sci Rep. 2016 Oct 10;6:34801. doi: 10.1038/srep34801 (PMC5056398; doi:10.1038/srep34801)
Supplement: Supplementary Information [file srep34801-s1.pdf]

Supplementary Information

Title:

Ecosystem responses to warming and watering in typical and desert steppes

Authors:

Zhenzhu Xu[1], Yanhui Hou[1], Lihua Zhang[1], Tao Liu[1,2] & Guangsheng Zhou[1,2]

[1]State Key Laboratory of Vegetation and Environmental Change, Institute of Botany, Chinese Academy of Sciences, Beijing 100093, China; [2]Chinese Academy of Meteorological Sciences, Beijing 100081, China.

Plate S1. Field infrared radiation warming facility in the typical (left) and desert (right) steppe ecosystems of North China.

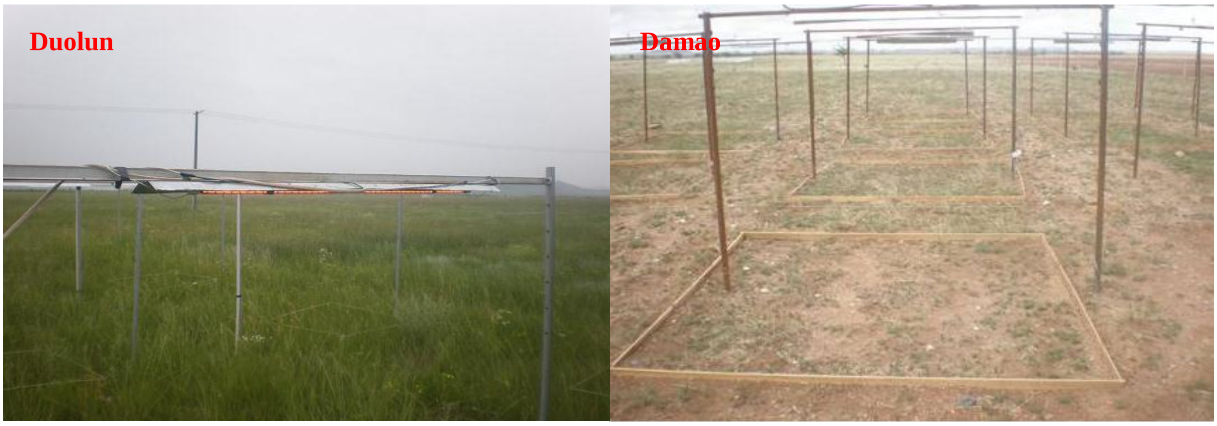

The photographs were taken by Zhenzhu Xu in 2012.

Figure S1. Changes in the mean annual precipitation (A, B) and in the annual averages of the daily mean, maximum, and minimum temperatures (C, D) over the past 60 years at the Duolun (A, C, 1953-2012) and Damao (B, D, 1955-2012) sites, Inner Mongolia, China. The meteorological data were collected by the Meteorological Stations of Duolun County, 26.0 km north-east of the typical steppe experimental site, and of Damao County, 13.5 km south-east of the desert steppe experimental site. The meteorological data were provided by the China Meteorological Data Sharing Service System.

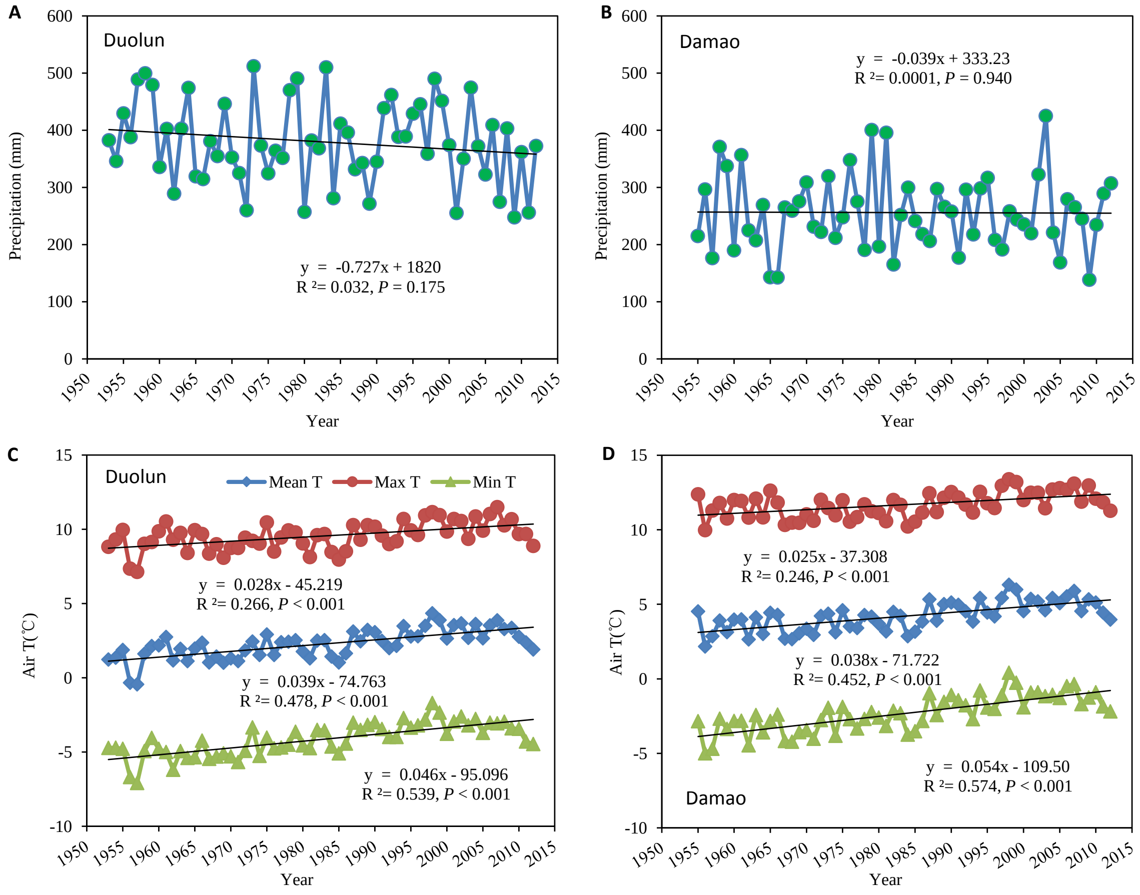

Figure S2. Relationship of the soil respiration rate with the chamber air temperature change (n = 183-454). We selected the air temperature in the soil chamber rather than the soil temperature because a best-fit exponential equation can be constructed using the air temperature for the semi-arid and arid areas ecosystems (similar to the desert measurement, Zhang and others 2009, Impact of temperature and soil water content on soil respiration in temperate deserts, China. Chin J Plant Ecol 33:936).

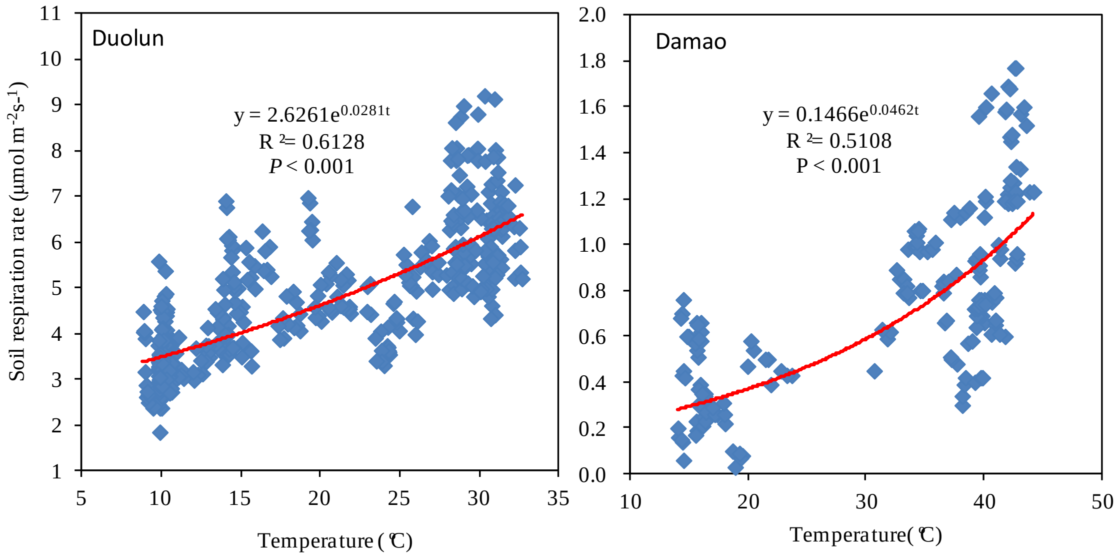

Figure S3. Relationships of the soil stoichiometric properties and the microbial viability with precipitation changes in the typical (left panels) and desert (right panels) steppes. For abbreviated details, see Table 1. Note the differences in the y-axis scales.

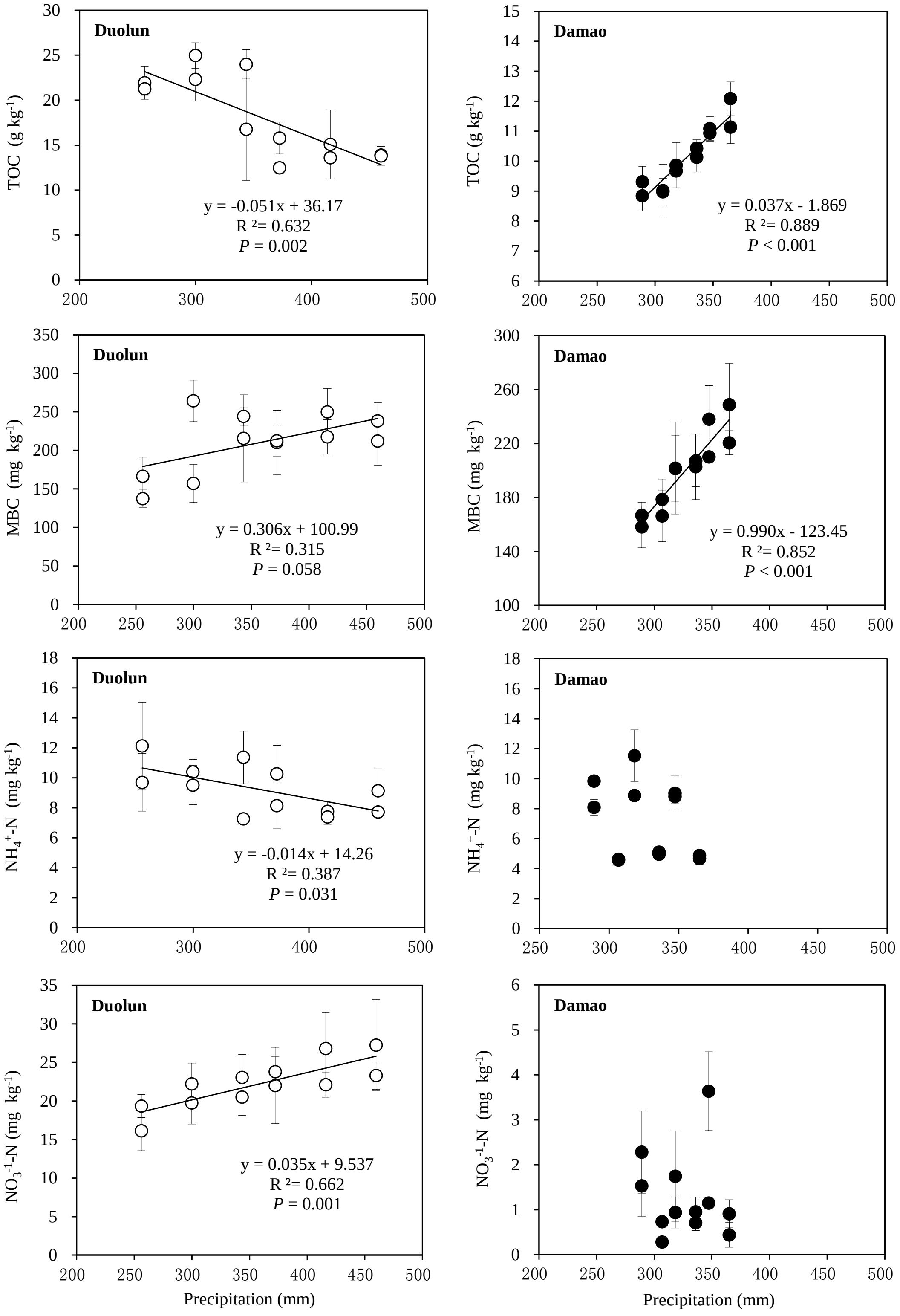

Table S1. A three-way ANOVA for the effects and their interactions on ANPP from ecosystem type, temperature, and watering.

Source	Sum of Squares	df	Mean Square	*F*	*P*
2011					
Corrected Model	41548.857	11	3777.169	2.368	0.030
Intercept	1035522	1	1035522	649.188	<0.001
Types	297.468	1	297.468	0.186	0.669
Temperatures	4.039	1	4.039	0.003	0.960
**Watering**	**26183.59**	**2**	**13091.79**	**8.207**	**0.001**
**Types × Temperatures**	**9403.776**	**1**	**9403.776**	**5.895**	**0.021**
Types × Watering	926.274	2	463.137	0.29	0.750
Temperatures × Watering	558.289	2	279.144	0.175	0.840
Types × Temperatures × Watering	2075.54	2	1037.77	0.651	0.529
Error	47853.08	30	1595.103		
Total	1141385	42			
Corrected Total	89401.94	41			
2012					
Corrected Model	138544.232	11	12594.93	45.222	<0.001
Intercept	2091519	1	2091519	7509.588	<0.001
**Types**	**98854.7**	**1**	**98854.7**	**354.937**	**<0.001**
Temperatures	166.023	1	166.023	0.596	0.448
**Watering**	**14003.42**	**2**	**7001.711**	**25.14**	**<0.001**
Types × Temperatures	55.131	1	55.131	0.198	0.660
**Types × Watering**	**24919.42**	**2**	**12459.71**	**44.737**	**<0.001**
Temperatures × Watering	392.519	2	196.26	0.705	0.504
Types × Temperatures × Watering	153.023	2	76.512	0.275	0.762
Error	6684.314	24	278.513		
Total	2236747	36			
Corrected Total	145228.5	35			

Bold font indicates significant effects of the factors or their interactions at $P < 0.05$, and × represents the interaction signs.

Table S2. A three-way ANOVA for the effects and their interactions on F_v/F_m from ecosystem type, temperature, and watering.

Source	Sum of Squares	df	Mean Square	*F*	*P*
Corrected Model	0.319	11	0.029	9.197	0.001
Intercept	261.893	1	261.893	83035.155	0.001
**Type**	**0.227**	**1**	**0.227**	**72.124**	**0.001**
Temperatures	0.001	1	0.001	0.274	0.601
**Watering**	**0.022**	**2**	**0.011**	**3.463**	**0.032**
**Type × Temperatures**	**0.014**	**1**	**0.014**	**4.505**	**0.034**
Type × Watering	0.008	2	0.004	1.334	0.264
Temperatures × Watering	0.01	2	0.005	1.576	0.208
Type × Temperatures × Watering	0.014	2	0.007	2.232	0.109
Error	1.429	453	0.003		
Total	288.894	465			
Corrected Total	1.748	464			

Bold font indicates significant effects of the factors or their interactions at $P < 0.05$, and × represents the interaction signs.

Table S3. A three-way ANOVA for the effects and their interactions on SR_{t20} from ecosystem type, temperature, and watering.

Source	Sum of Squares	df	Mean Square	*F*	*P*
2011					
Corrected Model	1226.419	11	111.493	302.652	<0.001
Intercept	1675.495	1	1675.495	4548.205	<0.001
**Types**	**1199.091**	**1**	**1199.091**	**3254.986**	**<0.001**
Temperatures	0.021	1	0.021	0.057	0.812
**Watering**	**2.556**	**2**	**1.278**	**3.47**	**0.033**
Types × Temperatures	0.033	1	0.033	0.088	0.767
Types × Watering	0.919	2	0.459	1.247	0.289
Temperatures × Watering	0.625	2	0.313	0.849	0.429
Types × Temperatures × Watering	1.476	2	0.738	2.004	0.137
Error	92.833	252	0.368		
Total	3477.889	264			
Corrected Total	1319.253	263			
2012					
Corrected Model	422.329	11	38.394	355.691	<0.001
Intercept	1268.122	1	1268.122	11748.3	<0.001
**Types**	**354.017**	**1**	**354.017**	**3279.731**	**<0.001**
**Temperatures**	**0.613**	**1**	**0.613**	**5.681**	**0.018**
**Watering**	**7.965**	**2**	**3.982**	**36.895**	**<0.001**
**Types × Temperatures**	**1.605**	**1**	**1.605**	**14.871**	**<0.001**
**Types × Watering**	**7.406**	**2**	**3.703**	**34.304**	**<0.001**
**Temperatures × Watering**	**1.924**	**2**	**0.962**	**8.913**	**<0.001**
**Types × Temperatures × Watering**	**5.531**	**2**	**2.765**	**25.619**	**<0.001**
Error	28.604	265	0.108		
Total	1490.782	277			
Corrected Total	450.934	276			

Bold font indicates significant effects of the factors or their interactions at $P < 0.05$, and × represents the interaction signs.

Table S4. A three-way ANOVA for the effects and their interactions on soil organic carbon (TOC) from ecosystem type, temperature, and watering.

Source	Sum of Squares	df	Mean Square	*F*	*P*
2011					
Corrected Model	1647.270a	11	149.752	7.876	<0.001
Intercept	10372.19	1	10372.19	545.541	<0.001
**Types**	1475.631	1	1475.631	77.613	**<0.001**
Temperatures	5.942	1	5.942	0.313	0.580
Watering	12.496	2	6.248	0.329	0.722
Types × Temperatures	2.975	1	2.975	0.156	0.695
Types × Watering	34.397	2	17.198	0.905	0.415
Temperatures × Watering	49.215	2	24.608	1.294	0.289
Types × Temperatures × Watering	44.696	2	22.348	1.175	0.323
Error	570.38	30	19.013		
Total	13977.736	42			
Corrected Total	2217.65	41			
2012					
Corrected Model	198.990a	11	18.09	1.994	0.066
Intercept	6089.312	1	6089.312	671.146	<0.001
**Types**	**144.793**	**1**	**144.793**	**15.959**	**<0.001**
Temperatures	6.152	1	6.152	0.678	0.417
Watering	10.099	2	5.05	0.557	0.579
Types × Temperatures	9.81	1	9.81	1.081	0.307
Types × Watering	14.678	2	7.339	0.809	0.455
Temperatures × Watering	8.965	2	4.483	0.494	0.615
Types × Temperatures × Watering	3.031	2	1.516	0.167	0.847
Error	272.19	30	9.073		
Total	6964.239	42			
Corrected Total	471.18	41			

Bold font indicates significant effects of the factors or their interactions at $P < 0.05$, and × represents the interaction signs.

Table S5. A three-way ANOVA for the effects and their interactions on microbial biomass carbon (MBC) from ecosystem type, temperature, and watering.

Source	Sum of Squares	df	Mean Square	*F*	*P*
2011					
Corrected Model	65670.381a	11	5970.035	2.197	0.043
Intercept	1593801.91	1	1593801.91	586.452	<0.001
Types	16.005	1	16.005	0.006	0.939
Temperatures	4737.062	1	4737.062	1.743	0.197
**Watering**	**35194.954**	**2**	**17597.477**	**6.475**	**0.005**
**Types × Temperatures**	**11542.253**	**1**	**11542.253**	**4.247**	**0.048**
Types × Watering	757.15	2	378.575	0.139	0.871
Temperatures × Watering	5550.35	2	2775.175	1.021	0.372
Types × Temperatures × Watering	2194.538	2	1097.269	0.404	0.671
Error	81531.067	30	2717.702		
Total	1775681.017	42			
Corrected Total	147201.449	41			
2012					
Corrected Model	24162.978	11	2196.634	0.848	0.597
Intercept	1909533.605	1	1909533.605	737.215	<0.001
Types	5228.688	1	5228.688	2.019	0.166
Temperatures	5.716	1	5.716	0.002	0.963
Watering	11597.469	2	5798.734	2.239	0.124
Types × Temperatures	2062.974	1	2062.974	0.796	0.379
Types × Watering	5291.379	2	2645.689	1.021	0.372
Temperatures × Watering	140.993	2	70.496	0.027	0.973
Types × Temperatures × Watering	861.169	2	430.584	0.166	0.848
Error	77706.016	30	2590.201		
Total	2080437.323	42			
Corrected Total	101868.994	41			

Bold font indicates significant effects of the factors or their interactions at $P < 0.05$, and × represents the interaction signs.

Table S6. A three-way ANOVA for the effects and their interactions on NH_4^+-N from ecosystem type, temperature, and watering.

Source	Sum of Squares	df	Mean Square	*F*	*P*
2011					
Corrected Model	83.906a	11	7.628	0.946	0.513
Intercept	3882.275	1	3882.275	481.478	<0.001
Types	4.756	1	4.756	0.59	0.448
Temperatures	1.073	1	1.073	0.133	0.718
Watering	7.373	2	3.687	0.457	0.637
Types × Temperatures	0.028	1	0.028	0.004	0.953
Types × Watering	8.774	2	4.387	0.544	0.586
Temperatures × Watering	32.75	2	16.375	2.031	0.149
Types × Temperatures × Watering	24.033	2	12.017	1.49	0.242
Error	241.897	30	8.063		
Total	4328.692	42			
Corrected Total	325.803	41			
2012					
Corrected Model	228.910a	11	20.81	3.586	0.003
Intercept	1903.036	1	1903.036	327.972	<0.001
**Types**	**163.03**	**1**	**163.03**	**28.097**	**<0.001**
Temperatures	10.762	1	10.762	1.855	0.183
Watering	8.006	2	4.003	0.69	0.509
Types × Temperatures	11.567	1	11.567	1.993	0.168
Types × Watering	14.146	2	7.073	1.219	0.310
Temperatures × Watering	4.954	2	2.477	0.427	0.656
Types × Temperatures × Watering	7.226	2	3.613	0.623	0.543
Error	174.073	30	5.802		
Total	2511.522	42			
Corrected Total	402.983	41			

Bold font indicates significant effects of the factors or their interactions at $P < 0.05$, and × represents the interaction signs.

Table S7. A three-way ANOVA for the effects and their interactions on NO_3^--N from ecosystem type, temperature, and watering.

Source	Sum of Squares	df	Mean Square	*F*	*P*
2011					
Corrected Model	3577.211	11	325.201	20.387	<0.001
Intercept	5003.063	1	5003.063	313.649	<0.001
**Types**	**3443.292**	**1**	**3443.292**	**215.864**	**<0.001**
Temperatures	5.025	1	5.025	0.315	0.579
Watering	35.653	2	17.827	1.118	0.340
Types × Temperatures	43.103	1	43.103	2.702	0.111
Types × Watering	31.232	2	15.616	0.979	0.387
Temperatures × Watering	2.597	2	1.299	0.081	0.922
Types × Temperatures × Watering	1.299	2	0.65	0.041	0.960
Error	478.535	30	15.951		
Total	10445.35	42			
Corrected Total	4055.746	41			
2012					
Corrected Model	5821.802	11	529.255	14.688	<0.001
Intercept	6387.238	1	6387.238	177.264	<0.001
**Types**	**5719.268**	**1**	**5719.268**	**158.726**	**<0.001**
Temperatures	2.889	1	2.889	0.08	0.779
Watering	9.403	2	4.701	0.13	0.878
Types × Temperatures	0.947	1	0.947	0.026	0.872
Types × Watering	6.593	2	3.296	0.091	0.913
Temperatures × Watering	29.369	2	14.685	0.408	0.669
Types × Temperatures × Watering	38.981	2	19.491	0.541	0.588
Error	1080.969	30	36.032		
Total	15305.071	42			
Corrected Total	6902.771	41			

Bold font indicates significant effects of the factors or their interactions at $P < 0.05$, and × represents the interaction signs.

Table S8. A significance list based on ANOVA for the effects and their interactions on the traits from ecosystem type, temperature, and watering.

Years	2011							2012					
Traits	ANPP	F_v/F_m	SR_{T20}	TOC	MBC	NH_4^+-N	NO_3^{-1}-N	ANPP	SR_{T20}	TOC	MBC	NH_4^+-N	NO_3^{-1}-N
E	ns	**	**	**	ns	ns	**	**	**	**	ns	**	**
T	ns	ns	Ns	ns	ns	ns	ns	ns	*	ns	ns	ns	ns
W	**	*	*	ns	**	ns	ns	**	**	ns	ns	ns	ns
E×T	*	*	ns	ns	*	ns	ns	ns	**	ns	ns	ns	ns
E×W	ns	ns	ns	ns	ns	ns	ns	**	**	ns	ns	ns	ns
T×W	ns	ns	ns	ns	ns	ns	ns	ns	**	ns	ns	ns	ns
E×T×W	ns	ns	ns	ns	ns	ns	ns	ns	**	ns	ns	ns	ns

Note: ** $P < 0.01$; *$P < 0.05$; ns, no significant differences; E, ecosystem types; T, temperatures; W, watering; ×, interaction signs; ANPP, annual aboveground net primary productivity; F_v/F_m, maximum photochemical efficiency of photosystem II; SR_{T20}, soil respiration at 20 ℃; TOC, soil total organic carbon; MBC, microbial biomass carbon.
